# *De Novo* Transcriptome Analysis of *Warburgia ugandensis* to Identify Genes Involved in Terpenoids and Unsaturated Fatty Acids Biosynthesis

**DOI:** 10.1371/journal.pone.0135724

**Published:** 2015-08-25

**Authors:** Xin Wang, Chen Zhou, Xianpeng Yang, Di Miao, Yansheng Zhang

**Affiliations:** CAS Key Laboratory of Plant Germplasm Enhancement and Specialty Agriculture, Wuhan Botanical Garden, Chinese Academy of Sciences, Wuhan, Hubei, China; Huazhong university of Science and Technology, CHINA

## Abstract

The bark of *Warburgia ugandensis* (Canellaceae family) has been used as a medicinal source for a long history in many African countries. The presence of diverse terpenoids and abundant polyunsaturated fatty acids (PUFAs) in this organ contributes to its broad range of pharmacological properties. Despite its medicinal and economic importance, the knowledge on the biosynthesis of terpenoid and unsaturated fatty acid in *W*. *ugandensis* bark remains largely unknown. Therefore, it is necessary to construct a genomic and/or transcriptomic database for the functional genomics study on *W*. *ugandensis*. The chemical profiles of terpenoids and fatty acids between the bark and leaves of *W*. *ugandensis* were compared by gas chromatography-mass spectrometry (GC-MS) analysis. Meanwhile, the transcriptome database derived from both tissues was created using Illumina sequencing technology. In total, about 17.1 G clean nucleotides were obtained, and *de novo* assembled into 72,591 unigenes, of which about 38.06% can be aligned to the NCBI non-redundant protein database. Many candidate genes in the biosynthetic pathways of terpenoids and unsaturated fatty acids were identified, including 14 unigenes for terpene synthases. Furthermore, 2,324 unigenes were discovered to be differentially expressed between both tissues; the functions of those differentially expressed genes (DEGs) were predicted by gene ontology enrichment and metabolic pathway enrichment analyses. In addition, the expression of 12 DEGs with putative roles in terpenoid and unsaturated fatty acid metabolic pathways was confirmed by qRT-PCRs, which was consistent with the data of the RNA-sequencing. In conclusion, we constructed a comprehensive transcriptome dataset derived from the bark and leaf of *W*. *ugandensis*, which forms the basis for functional genomics studies on this plant species. Particularly, the comparative analysis of the transcriptome data between the bark and leaf will provide critical clues to reveal the regulatory mechanisms underlying the biosynthesis of terpenoids and PUFAs in *W*. *ugandensis*.

## Introduction

*Warburgia ugandensis* Sprague, which belongs to a member of Canellaceae family, is a small evergreen tree distributed in eastern and southern Africa. *Warburgia* bark has been commonly used as traditional medicines for the treatment of gastro-intestinal disorders, cold, cough and sore throat, fever, malaria, respiratory and odontological problems in African countries [1]. It has been reported that the extract of *Warburgia* bark or leaf exhibits a variety of pharmacological effects, such as anti-bacterial, anti-fungal, anti-mycobacterial, antioxidant, and anti-inflammatory activities [2–6]. In addition, *W*. *ugandensis* is also used for fodder, insecticide, and toothbrush et al [1].

Several phytochemical studies reported the presence of diverse terpenoids in the bark and leaf of *W*. *ugandensis*, including waburganal, muzigadial, ugandenial A, and ugandensidial (cinnamodial) et al. [7–12]. The abundant terpenoids, especially those drimane- and colorotane-type sesquiterpenoides, were suggested to be relevant to its various medicinal effects [2, 3, 7, 12, 13]. Terpenoid is the largest class of plant natural products and plays crucial roles in plant growth development and ecological applications [14]. The biosynthesis of plant terpenoids can be divided into three stages as follows [15, 16]. In the first stage, the universal precursors, ispentenyldiphosphate (IPP) and dimethylallyl diphosphate (DMAPP), are formed via the 2-C-methyl-D-erythritol 4-phosphate (MEP) pathway and/or the mevalonate (MVA) pathway; second, geranyl diphosphate synthase (GPPS) and/or farnesyl diphosphate synthase (FPPS) convert the universal isoprenes to the terpenes precursors, geranyl diphosphate (GPP) and farnesyl diphosphate (FPP), respectively. In the last stage, the unique backbone of the terpenoids are generated under the catalysis of different kinds of terpenoid synthases (TPSs), including monoterpene and/or sesquiterpene synthases. Molecular characterization of TPSs has been intensively studied in many plant species, such as *Arabidopsis thaliana*, *Malus domestica*, *Oryza sativa*, and *Vitis vinifera* [17–21]. However, compared with the proceeding of chemical characterization of terpenoids, the study on the molecular characterization of TPSs, the terpenoid-forming enzymes, is far away backward. For example, in *W*. *ugandensis*, although several terpenoids have been structurally characterized, there is no TPS characterized in this plant at molecular level so far.

Polyunsaturated fatty acids (PUFAs) are a class of fatty acids that contain two or more double bonds in their backbones. Some PUFAs such as linoleic acid (18:2) and linolenic acid (18:3) cannot be synthesized in mammals or humans, and human only consumes them through the diet. PUFAs, especially those omega-3 (ω-3) types, are important for maintaining human body’s normal metabolism and provide numerous health benefits, including anticancer properties, prevention and treatment of coronary heart disease, hypertension and type 2 diabetes[22, 23]. It has been reported that the crude extract of *W*. *ugandensis* shows anti-mycobacterial activity and its stem bark can be used to treattuberculosis, and these effects are probably due to the presence of linoleic acid (18:2) and drimane sesquiterpenoids [7]. In plant, fatty acids are generally synthesized from acetyl-CoA in a three-step process to form palmitic acid (16:0) or stearic acid (18:0). Then, stearic acid (18:0) is desaturated to oleic acid (18:1), linoleic acid (18:2), or linolenic acid (18:3) by specific fatty acid desaturases (FADs), including FAD2 and FAD3, which are key enzymes to control the biosynthesis of unsaturated fatty acids [24]. Finally, linoleic acid (18:2) or linolenic acid (18:3) can be further converted into a variety of complex PUFAs under the catalysis of a series of desaturases and elongases [25, 26].

In recent years, due to the high-throughput, accuracy, and reproducibility, mRNA sequencing (RNA-Seq) technology has emerged as a powerful tool to profile the genome-wide transcriptional pattern in different tissues and/or at different developmental stages [27], and discover novel genes in specific biological processes, especially in those non-model organisms without genomic sequences [28–30]. To date, the genomic information of *W*. *ugandensis* is not available yet, and only 20 nucleotide sequences (or ESTs), including five partial cDNAs encoding for putative sesquiterpene synthases, have been deposited in the NCBI GenBank database. Considering the medicinal and economic importance of *W*. *ugandensis*, it deserves to lunch genomic studies on this plant species, especially to investigate the biosynthesis of its medicinal components, e.g. terpenoids and PUFAs. To start this project, we reported here the construction of *W*. *ugandensis* transcriptome database derived from its leaf and bark, which are the tissue sources for its medical use. As a result, a total of 17.1 G clean nucleotides were obtained and *de novo* assembled into 72,591 unigenes thereafter. This database will provide an important resource in identifying genes encoding enzymes in terpenoids and PUFAs biosynthetic pathways in *W*. *ugandensis*.

## Results

### Chemical analysis of terpenoids and fatty acids in the leaf and bark of *W*. *ugandensis*

**Fig 1:**
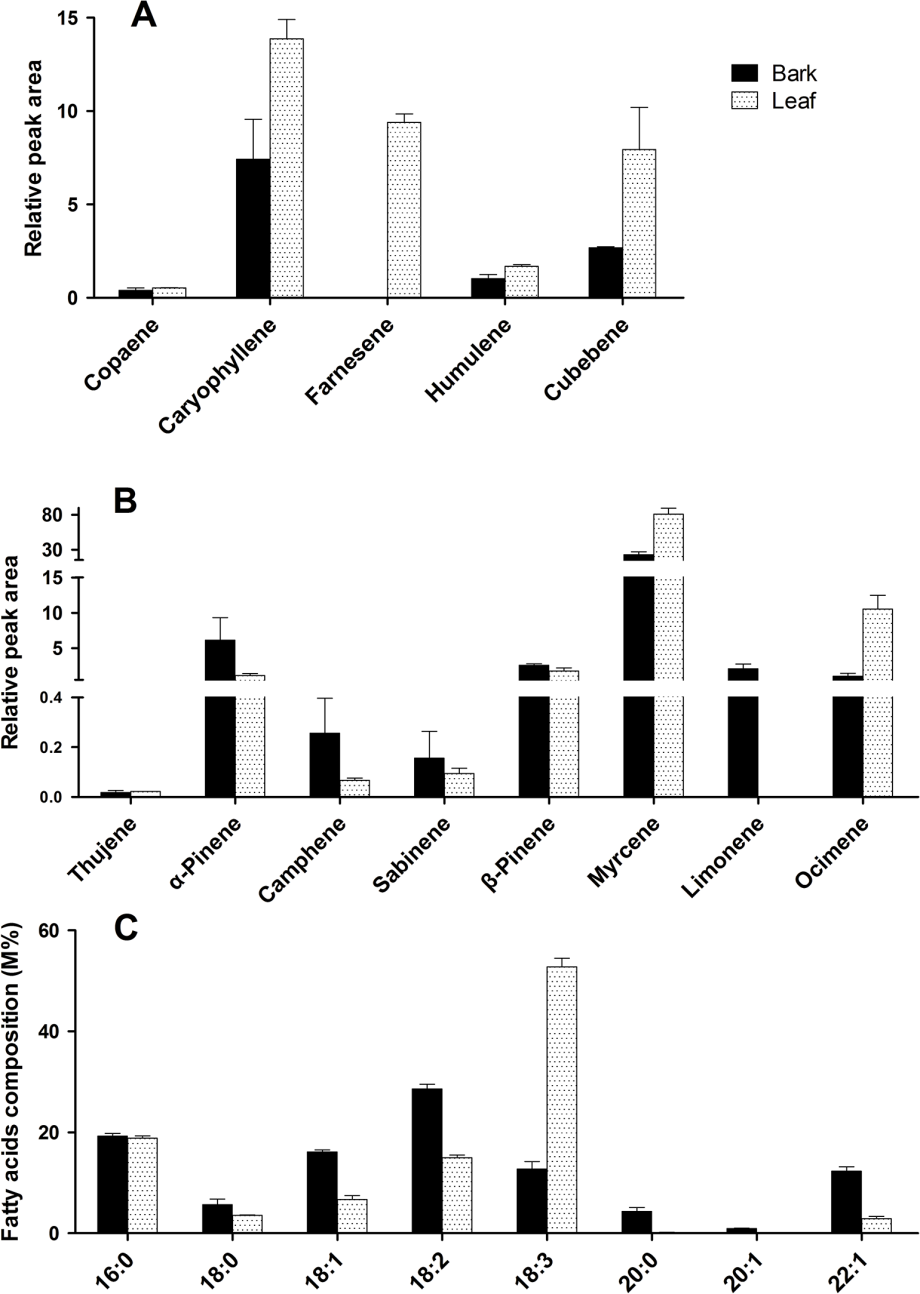
The phytochemical analysis of terpenoid and fatty acid compositions in *W*. *ugandensis* bark and leaf. The relative content of monoterpenes (A), sesquiterpenes (B), and fatty acid compositions (C) were shown.

Since terpenoids and PUFAs are thought to be potential bioactive compounds of *W*. *ugandensis*, those compounds were analyzed and compared between its bark and leaf. As shown in Fig 1A, five sesquiterpenoids, including copaene, caryophyllene, farnesene, humulene, and cubebene, were detected by gas chromatography-mass spectrometry (GC-MS). Except that farnesene is exclusively synthesized in the leaf, the other four sesquiterpenoids are presented in both tissues with their concentrations being relatively higher in the leaf. Eight monoterpenoids were detected by this GC-MS analysis. The content of myrcene and ocimene is significantly higher in the leaf than that in the bark, while more of other monoterpenoids such as α-pinene and camphene is detected in the bark, especially limonene is only found in the bark but not in the leaf (Fig 1B).

PUFAs consist of the major proportion of fatty acids in *W*. *ugandensis* bark and leaf, accounting for 41.34% and 67.76% of their total fatty acids, respectively (Fig 1C). In the bark, the most abundant of PUFA is linoleic acid (18:2), which accounts for 28.59% of the total fatty acids; while linolenic acid (18:3) is the highest amount of PUFA in the leaf, reaching up to 52.77% of the total fatty acids. Palmitic acid (16:0) accounts for roughly equal proportions (about 19%) in the both tissues. The remaining types of fatty acids, such as stearic acid (18:0), oleic acid (18:1), and erucic acid (22:1), are detected in a higher concentration in the bark relative to the leaf.

### Transcriptome sequencing and *de novo* assembly

Two cDNA libraries from the bark and leaf of *W*. *ugandensis* were constructed by the Illumina sequencing technology, which produced 88,241,216 and 85,560,090 raw reads in the bark and leaf, respectively (Table 1). After removing the low-quality reads and adapter sequences, 87,093,692 clean reads for the bark and 84,389,152 clean reads for the leaf were obtained; and then a total of 17.1 G data were further *de novo* assembled into 72,591 unigenes with an average size of 1,051 bp and an N50 of 1,467 bp. The length distribution of the *W*. *ugandensis* unigenes showed that approximately 31% of the unigenes was presented in a length of more than 1 Kb (S1 Fig). These results showed that the throughout and sequencing quality were high enough for further bioinformatics analyses.

**Table 1:** Statistic of sequencing and *de novo* assembling of transcriptome in *W*. *ugandensis*.

Item	Sample	Number (n)	Sequence (bp)	Valid ratio (%)	GC (%)	Q30 (%)	N50 (bp)	Average length (bp)
raw read	Leaf	88241216	8824121600	—	—	—	—	—
	Bark	85560090	8556009000	—	—	—	—	—
clean read	Leaf	87093692	8693658441	98.52	45.00	88.89	—	—
	Bark	84389152	8422552230	98.44	45.50	89.38	—	—
Total	Unigenes	72591	76284840	—	—	—	1467	1050.89

### Functional annotation and classification of unigenes

For functional annotation of the *W*. *ugandensis* transcriptome, the unigenes were searched against diverse databases using BLASTX program. Only 38.06% of unigenes (27,631) were annotated in the NCBI non-redundant (Nr) protein database at a cut-off E-value of 10^−5^ (Table 2). The relative low number of annotated unigenes was probably due to no publicly available genome or the limitations of EST information for species in the Canellaceae family. Out of these hits, 16,366 unigenes (59.23%) had an E-value of less than 10^−45^, indicating they had very strong similarities to known proteins.

**Table 2:** Statistics of annotations for assembled unigenes in *W*. *ugandensis*.

Database	Account	Percentage (%)^a^
Nr	27631	38.06
SWISS-PROT	20145	27.75
KOG	16636	22.92
GO	21702	29.90
KEGG	6489	8.94
Total	27995	38.6

^a^ The percent of annotated unigenes in the total 72591 assembled unigene.

**Fig 2:**
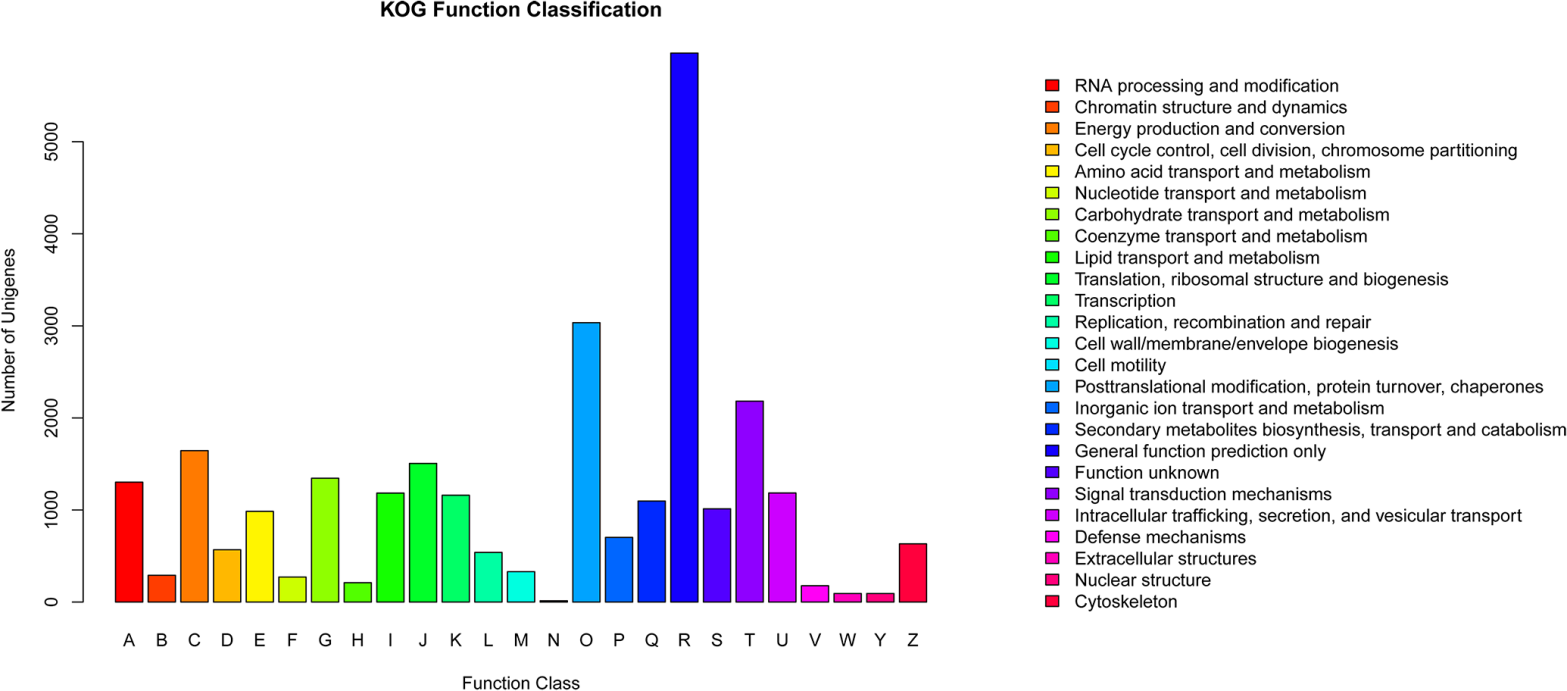
KOG function classification of unigenes from the *W*. *ugandensis* transcriptome. A total of 72,591 unigenes were assigned to 25 subclasses.

The KOG database refers Cluster of Orthologous Groups of gene products. Every protein in the KOG is assumed to be evolved from an ancestor protein, and thus they are either orthologs or paralogs. The KOG annotation showed that 16,636 (22.92%) unigenes were classified into 25 KOG categories. The cluster for “General function prediction only” represented the largest group, followed by “posttranslational modification, protein turnover, chaperones”, and “signal transduction mechanisms” (Fig 2). Additionally, 1097 unigenes (6.59% of total KOG hits) were categorized into “secondary metabolites biosynthesis, transport and catabolism”.

**Fig 3:**
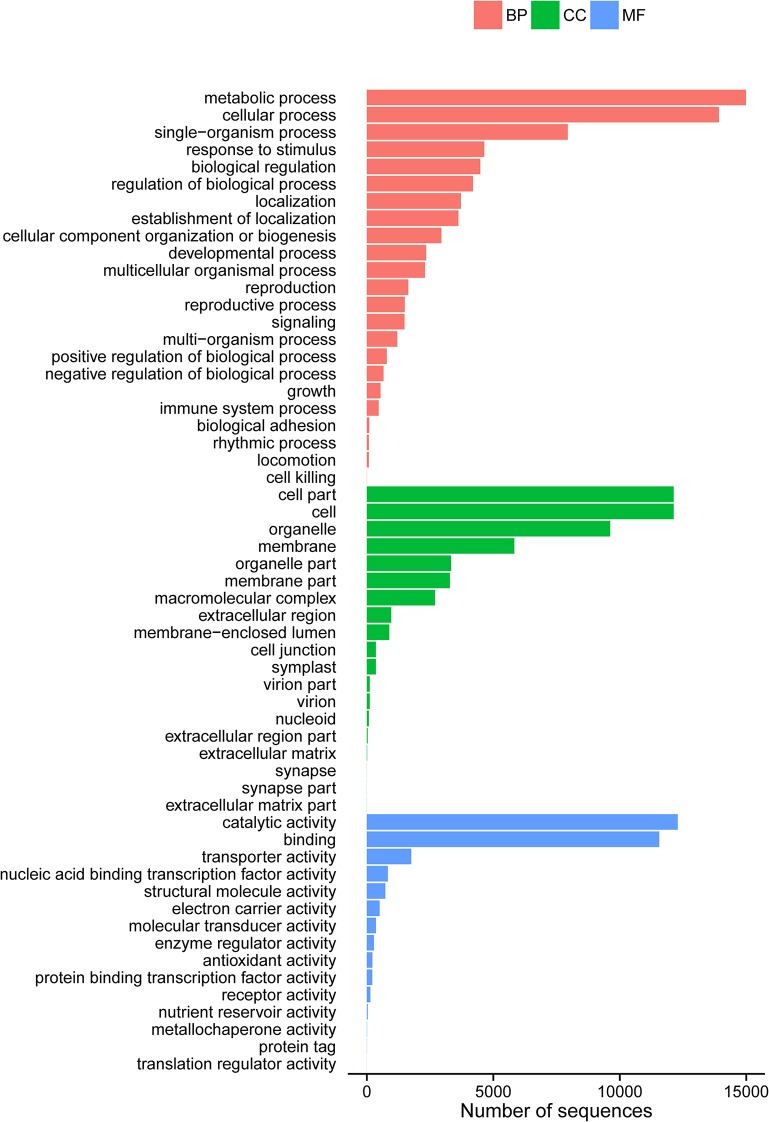
Histogram of the gene ontology classifications of annotated unigenes from the *W*. *ugandensis* transcriptome. BP, Biological process; CC, Cell component; MF, Molecular function.

To further functionally categorize the *W*. *ugandensis* transcriptome, a total of 21,702 (29.90%) of the Nr-annotated unigenes were assigned into different GO terms according to their associated biological processes, cellular components and molecular function. In the “biological process” category, the term “metabolic process” and “cellular processes” were predominant; and in the “cellular component” group, “cell parts” and “cell” were found to be the largest classes; while under the “molecular function” class, the majority of unigenes were assigned to be involved in “catalytic activities” and “binding” (Fig 3).

To better understand the function of the assemble unigenes in biological pathways; they were searched against the KEGG pathway database. It was found that only 6,489 (8.94%) unigenes were assigned to 327 KEGG pathways (See S1 Table). The highest represented pathway was “carbon metabolism” (ko01200, 550 unigenes), followed by “ribosome” (ko03010, 510 unigenes) and “biosynthesis of amino acids” (ko01230, 349 unigenes). Since sesquiterpenoids and PUFAs were thought to be medicinal components of *W*. *ugandensis*, we focused our efforts to survey the unigenes whose functions related to terpenoid and fatty acid metabolism (Table 3). As a result, 53 unigenes were identified for “terpenoid backbone biosynthesis” (ko00900), 20 unigenes for “sesquiterpenoid and triterpenoid biosynthesis” (ko00909), 13 unigenes for “monoterpenoid biosynthesis” (ko00902), 94 unigenes for “fatty acid biosynthesis” (ko00061), and 100 unigenes for “biosynthesis of unsaturated fatty acids” (ko01040).

**Table 3:** Summary of unigenes related to lipid and terpenoid metabolism.

Pathway type	Pathway ID	Pathway Name	Unigene Number
Fatty acid metabolism	ko00061	Fatty acid biosynthesis	94
	ko00062	Fatty acid elongation	27
	ko00071	Fatty acid degradation	119
	ko00591	Linoleic acid metabolism	14
	ko00592	Linolenic acid metabolism	55
	ko01040	Biosynthesis of unsaturated fatty acids	100
Terpenoids metabolism	ko00900	Terpenoid backbone biosynthesis	53
	ko00902	Monoterpenoid biosynthesis	13
	ko00904	Diterpenoid biosynthesis	10
	ko00909	Sesquiterpenoid and triterpenoid biosynthesis	20

### Putative structural genes involved in terpenoid backbone biosynthesis pathway

**Fig 4:**
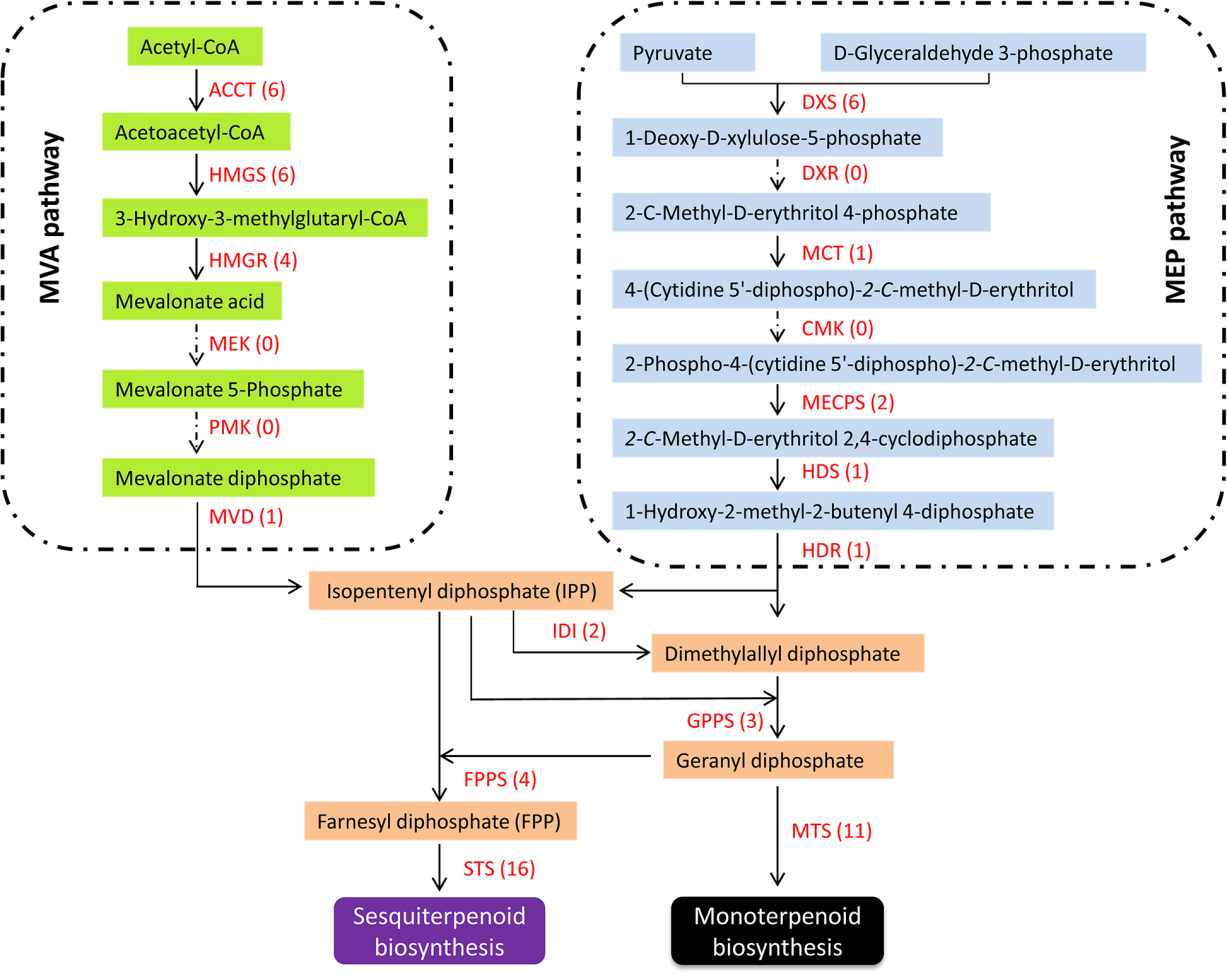
Putative structural genes involved in terpenoid backbone biosynthesis pathway. The values in the bracket indicate the number of unigenes in the corresponding gene families. ACCT, acetyl-CoA C-acetyltransferase; HMGS, hydroxymethylglutaryl-CoA synthase; HMGR, hydroxymethylglutaryl-CoA reductase; MVK, mevalonate kinase; PMK, phosphomevalonate kinase; MVD, mevalonate diphosphate decarboxylase; DXS, 1-deoxy-D-xylulose-5-phosphate synthase; DXR, 1-deoxy-D-xylulose-5-phosphate reductoisomerase; MCT, 2-C-methyl-D-erythritol 4-phosphate cytidylyltransferase; CMK, 4-diphosphocytidyl-2-C-methyl-D-erythritol kinase; MECPS, 2-C-methyl-D-erythritol 2,4-cyclodiphosphate synthase; HDR, 4-hydroxy-3-methylbut-2-enyl diphosphate reductase; HDS, (E)-4-hydroxy-3-methylbut-2-enyl-diphosphate synthase; IDI, isopentenyl-diphosphate delta-isomerase; GPPS, geranyl diphosphate synthase; FPPS, farnesyl diphosphate synthase; STS, Sesquiterpenoid synthase; MTS, Monoterpenoid synthase.

Although mono- and sesqui-terpenoids were detected in *W*. *ugandensis*, the structural genes involved in these biosynthetic pathways have been largely unknown so far. Analysis of the transcriptome data allowed us to identify 17 unigenes coding for four enzymes in the mevalonate pathway and 20 unigenes for five enzymes in the MEP pathway, which synthesizes the common precursor isopentenyl diphosphate (IPP) for downstream terpenoid pathways (Fig 4). Like those in other organisms, the structural genes for upstream terpenoid pathways are presented as multi-members in *W*. *ugandensis*. For example, six unigenes were predicted to code for hydroxymethylglutaryl-CoA reductase (HMGR), which is the first committed enzyme in the mevalonate pathway to form mevalonate (S2 Table); six unigenes were for 1-deoxy-D-xylulose-5-phosphate synthase (DXS) that catalyzes the conversion of pyruvate and D-glyceraldehyde 3-phosphate into 1-deoxy-D-xylulose-5-phosphate (DOXP) in the first step in the MEP pathway; two unigenes were for isopentenyl-diphosphate delta-isomerase (IDI), which catalyzes the isomerization of isopentenyl diphosphate to dimethylallyl diphosphate; seven unigenes were annotated to encode prenyltransferases in terpenoid pathways, of which three unigenes were for GPPSs and four unigenes for FPPSs.

### Identification of putative TPSs in the *W*. *ugandensis* transcriptome

**Fig 5:**
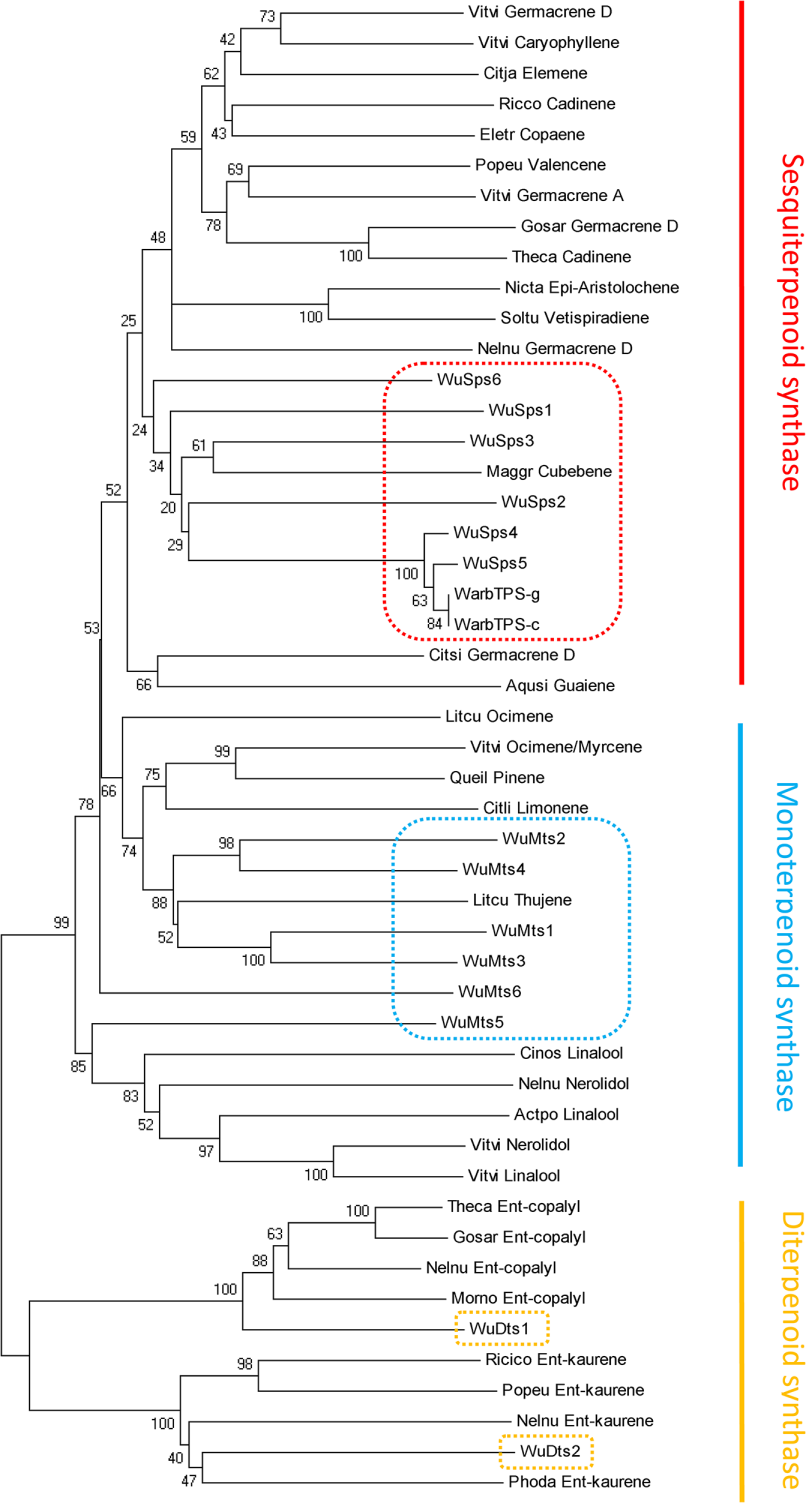
Phylogenetic tree of terpene synthases. Phylogenetic analysis of 14 putative *W*. *ugandensis* WuTPS protein sequences with their homologs from other plants indicates that they are clustered into three main clades including: monoterpenoid synthase (WuMts), sesquiterpenoid synthase (WuSps), and diterpenoid synthase (WuDts). WarbTPS-c (ACJ46047.1, putative sesquiterpene synthase, *W*. *ugandensis*); WarbTPS-g (ACJ46048.1, putative sesquiterpene synthase, *W*. *ugandensis*); WuSps1, CL29873Contig1; WuSps2, CL29511Contig1; WuSps3, CL3178Contig1; WuSps4, CL4160Contig1; WuSps5, comp68897_c0_seq1; WuSps6, CL24969Contig1; WuSps7, CL30258Contig1; WuMts1, CL27268Contig1; WuMts2, CL27339Contig1; WuMts3, CL30385Contig1; WuMts4, CL276Contig2; WuMts5, CL1Contig9269; WuMts6, CL29966Contig1; WuMts7, CL14869Contig1; WuDts1, CL9128Contig1; WuDts2, CL28648Contig1; Citsi_Germacrene_D (XP_006494713.1, (-)-germacrene D synthase-like isoform X2, *Citrus sinensis*); Popeu_Valencene (XP_011015484.1, valencene synthase-like, *Populus euphratica*); Nelnu_Germacrene_D (XP_010258444.1, (-)-germacrene D synthase-like, *Nelumbo nucifera*); Eletr_Copaene (ADK94034.1, alpha-copaene synthase, *Eleutherococcus trifoliatus*); Gosar_Germacrene_D (KHG04103.1, (-)-germacrene D synthase, *Gossypium arboretum*); Vitvi_Germacrene_D (XP_010644711.1, (-)-germacrene D synthase, *Vitis vinifera*); Vitvi_Germacrene_A (ADR66821.1, Germacrene A synthase, *Vitis vinifera*); Citja_Elemene (BAP74389.1, delta-elemene synthase, *Citrus jambhiri*); Theca_Cadinene (EOY12648.1, Delta-cadinene synthase isozyme A, *Theobroma cacao*); Ricco_Cadinene (EEF38721.1, (+)-delta-cadinene synthase isozyme A, *Ricinus communis*); Aqusi_Guaiene (AIT75875.1, putative delta-guaiene synthase, *Aquilaria sinensis*); Vitvi_Caryophyllene (AEP17005.1, (E)-beta-caryophyllene synthase, *Vitis vinifera*); Maggr_Cubebene (ACC66281.1, beta-cubebene synthase, *Magnolia grandiflora*); Cinos_Linalool (AFK09265.1, S-(+)-linalool synthase, *Cinnamomum osmophloeum*); Nelnu_Nerolidol (XP_010248179.1, (3S,6E)-nerolidol synthase 1-like, *Nelumbo nucifera*); Vitvi_Nerolidol (XP_010646919.1, (3S,6E)-nerolidol synthase 1, chloroplastic-like isoform X1, *Vitis vinifera*); Vitvi_Linalool (ADR74212.1, (3S)-linalool/(E)-nerolidol synthase, *Vitis vinifera*); Actpo_Linalool (ADD81295.1, linalool synthase, *Actinidia polygama*); Nelnu_Ent-copalyl (XP_010277558.1, ent-copalyl diphosphate synthase, chloroplastic-like, *Nelumbo nucifera*); Theca_Ent-copalyl (XP_007050589.1, Copalyl diphosphate synthase, *Theobroma cacao*); Morno_Ent-copalyl (XP_010090409.1, Ent-copalyl diphosphate synthase, *Morus notabilis*); Gosar_Ent-copalyl (KHG01750.1, Ent-copalyl diphosphate synthase, chloroplastic, *Gossypium arboreum*); Nelnu_Ent-kaurene (XP_010260722.1, ent-kaur-16-ene synthase, chloroplastic isoform X1, *Nelumbo nucifera*); Phoda_Ent-kaurene (XP_008809130.1, ent-kaur-16-ene synthase, chloroplastic, *Phoenix dactylifera*); Ricico_Ent-kaurene (XP_002533694.1, Ent-kaurene synthase B, chloroplast precursor, *Ricinus communis*); Popeu_Ent-kaurene (XP_011014299.1, ent-kaur-16-ene synthase, chloroplastic, *Populus euphratica*); Nicta_Epi-Aristolochene (3M02.A, 5-Epi-Aristolochene Synthase, *Nicotiana tabacum*); Soltu_Vetispiradiene (Q9XJ32.1, vetispiradiene synthase 1, *Solanum tuberosum*); Litcu_Ocimene (AEJ91554.1, trans-ocimene synthase, *Litsea cubeba*); Litcu_Thujene (AEJ91555.1, alpha-thujene synthase, *Litsea cubeba*); Citli_Limonene (AAM53946.1, (+)-limonene synthase 2, *Citrus limon*); Vitvi_Ocimene/Myrcene (ADR74206.1, (E)-beta-ocimene/myrcene synthase, *Vitis vinifera*); Queil_Pinene (CAK55186.1, pinene synthase, *Quercus ilex*).

TPSs are the primary enzymes responsible for catalyzing the conversion of prenyl diphosphates (GPP or FPP) into monoterpenes (C10), sesquiterpenes (C15), or diterpenes (C20). The multiple diversities of terpene carbon skeletons can be largely attributed to a great number of different TPSs [15]. The PFAM motif PF01397 (N-terminal TPS domain) [31] was used to search against the assembled *W*. *ugandensis* unigenes. As a result, a total of 16 unigenes were found to encode putative TPS, of which 9 unigenes were predicted to contain full-length coding sequences (S3 Table). To infer their possible functions and better describe the evolutionary relationships, phylogenetic analysis of the putative TPSs protein sequences and their homologs in other plants was performed, which indicated that they were divided into three main clades (Fig 5). In the first group, seven TPSs (WuSps1-7) are likely to be involved in sesquiterpene biosynthesis, which are clustered with two available *W*. *ugandensis* TPSs sequences (WargTPS-c, ACJ46047.1; WargTPS-g, ACJ46048.1) and a *β*-cubebene synthasefrom *Magnolia grandiflora* (ACC66281.1) in the same subclass. Seven unigenes (WuMts1-7) were found to be probably involved in monoterpene biosynthesis, and scattered into different subgroups. For example, four WuMts (WuMts1-4) are clustered with a *Litsea cubeba* thujene synthase (AEJ91555.1), while WuMts5 and WuMts7 forms a separate subgroup with several linalool- or nerolidol synthase homologs from other plants. The remaining two TPSs (WuDts1 and WuDts2) with full-length sequence were predicted to participate in diterpene biosynthesis, exhibiting closer relationships with several ent-copalyl diphosphate synthases and ent-kaurene synthases.

### Putative genes related to unsaturated fatty acids metabolism

The high pharmaceutical effects of *W*. *ugandensis* were reported to be associated to its ability to biosynthesize considerable amounts of PUFAs including linoleic acid (18:2) [1, 7, 8]. To gain a better understanding of the mechanisms underlying PUFAs biosynthesis, we summarized the unigenes related to unsaturated fatty acids metabolism pathway in the database. For identification of genes in unsaturated fatty acid metabolic pathway, the assemble unigenes were searched against the KEGG pathway database. As a result, 100 genes were identified for “biosynthesis of unsaturated fatty acids” pathway (ko01040), most of which appeared to encode enzymes responsible for fatty acid desaturation and chain elongation (Table 3, S4 Table). For example, we identified nine unigenes encoding putative 3-oxoacyl-[acyl-carrier-protein] reductase (EC 1.1.1.100), which is a key enzyme participated in PUFAs biosynthesis, catalyzing the conversion of 3-hydroxyacyl-[acyl-carrier-protein] into 3-oxoacyl-[acyl-carrier-protein] with NAD^+^ or NADP^+^ as acceptor. In addition, 21 genes were found to encode putative fatty acid desaturases, of which 13 unigenes were annotated as stearoyl-CoA desaturase, the enzyme catalyzing the removal of two hydrogen atoms of stearic acid (18:0) to form oleic acid (18:1), seven unigenes were predicted to code for delta-12fatty acid desaturase (or ω-6 fatty acid desaturase), a key enzyme in converting oleic acid (18:1) into linoleic acid (18:2), and one unigene was predicted to encode ω-3 fatty acid desaturase, the enzyme catalyzing linoleic acid (18:2) to form linolenic acid (18:3).

### Differentially expressed unigenes between *W*. *ugandensis* bark and leaf

To identify the differentially expressed genes (DEGs) between the bark and leaf, the FPKM (fragments per kb per million reads) method was used to calculate the expression levels of the unigenes. The results showed that 12,704 unigenes were specifically expressed in the bark while 2,420 unigenes were specific for the leaf(S2 Fig). A total of 2,324 unigenes were expressed in both tissues but their expressions were dramatically different between the leaf and bark (with fold changes larger than two and *p*≤0.05). Among these DEGs, 818 unigenes were up-regulated, while 1,506 were down-regulated in the *W*. *ugandensis* bark as compared to the leaf (S2 Fig and S5 Table). GO enrichment analysis of the DEGs indicated that a total of 302 GO terms were significantly enriched as compared to the genomic background (S6 Table). Additionally, 15 metabolic pathways were significantly over-represented by the KEGG pathway enrichment analysis (S7 Table). Among them, photosynthesis (ko00195) process was the most enriched pathway, and the remaining pathways were mainly related to secondary metabolites biosynthesis including monoterpenoid biosynthesis (ko00902), phenylpropanoid biosynthesis (ko00940), and novobiocin biosynthesis (ko00401), and fatty acid metabolism (cutin, suberine and wax biosynthesis, ko00073; fatty acid elongation, ko00062).

### Quantitative real-time reverse transcription PCR (qRT-PCR) analysis of DEGs

**Fig 6:**
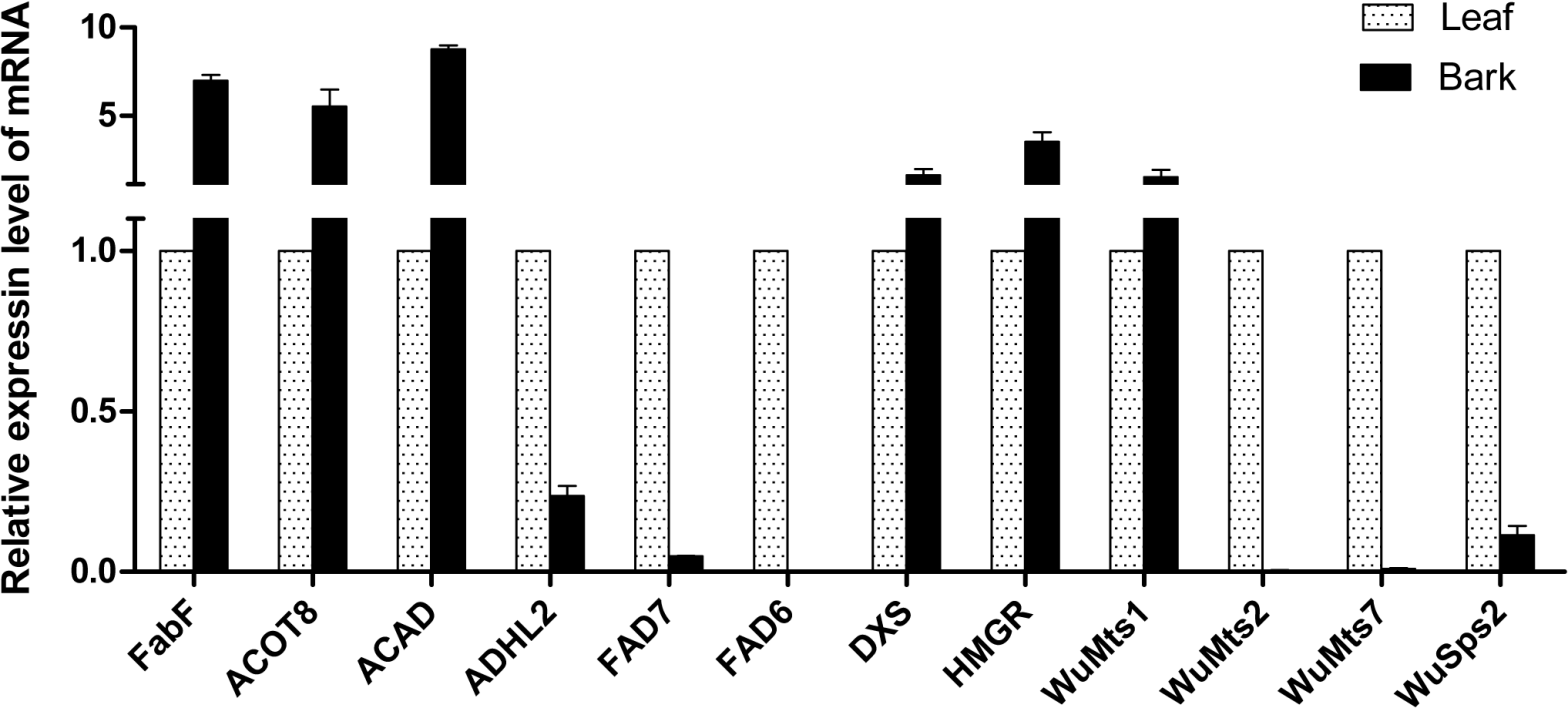
qRT-PCR analysises. qRT-PCRwas performed to validate the 12 differentially expressed unigenes related to terpenoid biosynthesis and/or unsaturated fatty acid metabolisms. The gene names, serial numbers and primer sequences used for qRT-PCR analysis are shown in S8 Table.

To confirm the difference in expression of unigenes detected by RNA-seq, 12 DEGs that were predicted to be coding for key enzymes involved in terpenoid and unsaturated fatty acid metabolic pathways were selected for qRT-PCR validation (S8 Table). As a result, six unigenes exhibited higher expression levels in the bark than those in the leaf, including FabF, ACOT8, ACAD, DXS, HMGR and WuMts1; while the remaining six unigenes, such as FAD7 (a ω-3 fatty acid desaturase) and FAD6 (a ω-6 fatty acid desaturase) showed lower expression levels in the bark than those in the leaf (Fig 6). The data of qRT-PCRs mostly match the results by RNA-seq, which suggests the reliability of the transcriptome database (S8 Table).

## Discussion

Pharmacological studies on *W*. *ugandensis* have indicated that sesquiterpenoids and PUFAs are active compounds for its medical use [2].The bark of the plant is mostly used for its medical treatments while its leaf is also utilized in some cases [1]. These literatures drove us to compare the chemical profiles of terpenoids and PUFAs between the bark and leaf. Five sesquiterpenoids (copaene, caryophyllene, farnesene, humulene, and cubebene) and two monoterpenoids (myrcene and ocimene) were detected on the condition of our GC-MS analysis. Unexpectedly, their contents are significantly higher in the leaf relative to the bark (Fig 1). On the other hand, the total proportion of PUFAs in the leaf also showed a little higher level than that in the bark. Due to over-exploitation of the bark of the plant for its medical use, the population of the *Warburgia* species has considerably declined throughout their natural habitats [32]. The phytochemical study of *W*. *ugandensis* here hinted that its leaf might be also an important resource for the active compounds and could be used as a good replacement for bark, as the higher yield of the leaf holds great potential for its pharmacological applications [33].

In recent years, RNA-seq has been applied to several medicinal plants without available genomic information, such as *Eugenia uniflora* L. and *Litsea cubeba* [28, 30]. Before we lunch this project, little genomic information and very few ESTs sequences of *W*.*ugandensis* are available in public databases. Here, *de novo* transcriptome analysis of *W*. *ugandensis* bark and leaf was performed using the Illumina sequencing platform. This work will provide an important public genomic resource and facilitate further functional genomic studies on this plant.

Terpenoid biosynthetic pathway has been well studied, and many genes in this pathway have been identified in other plants [17, 28, 34, 35]. In this work, a total of 37 unigenes were found to be participated in terpenoid backbone biosynthesis, which showed high similarity to those homologs from other plants. For downstream terpenoid pathways, 14 putative TPSs were found in the *W*. *ugandensis* transcriptome, the number of the identified TPSs seemed to be reasonable in medicinal plants. For example, 14 TPSs in *L*. *cubeba* and 17 TPSs in *T*.*laciniata* were identified by use of *de novo* RNA sequencing platform [20, 30]. Phylogenic tree analysis of the identified TPSs showed that most of them clustered with WuSps (WuSps1-6 represent sesquiterpene synthases) and WuMts (WuMts1-6 represent monoterpene synthases), which correlates well with the fact that sesquiterpenes and monoterpenes are the major terpenoids in *W*. *ugandensis*. For the pathway of PUFAs, it has been well dissected in some oil plants by RNA-seq strategy [36–38]. Fatty acid desaturation and long chain elongation are the key processes in PUFAs formation. In this study, 21unigenes encoding putative fatty acid desaturases and seven unigenes for long chain elongases are present in the *W*.*ugandensis* database (S4 Table). Similarly, 21 unigenes coding for fatty acid desaturases were previously identified in *Sacha Inchi* by RNA-seq [37].

Comparative transcriptome analysis showed that most of DEGs were enriched in the photosynthesis, followed by several secondary metabolism pathways, including phenylalanine metabolism and phenylpropanoid biosynthesisthat provide the precursors for lignin formation. This result was consistent with the fact that photosynthesis reactions often take place in leaf, while lignin mostly accumulates in bark. In the datasets, it was found that seven out of eight WuSps (except for WuSps4) had higher expression levels in the leaf than in the bark according to their FPKM values (S3 Table), which correlates well with our phytochemical results, in which higher concentrations of sesquiterpenoids were detected in the leaf than in the bark (Fig 1A). For example, both WuSps2 and WuSps7, exhibiting highly homologous to a beta-cubebene synthase from *M*. *grandiflora*, were found to be expressed at significantly higher levels in the leaf than in the bark, which might result in a higher level of cubebene accumulated in its leaf. Furthermore, the chemical profiles indicated that linoleic acid (18:2) and linolenic acid (18:3) are the major constituents of PUFAs in *W*. *ugandensis*, but their proportions are very distinct between the leaf and bark, which is probably due to the difference in the expression of fatty acid desaturases in PUFAs biosynthesis. Among the 21 putative fatty acid desaturases in the database, the expression levels of *FAD6* and *FAD7* were significantly higher in the leaf than in the bark. FAD6 is the enzyme to desaturate oleic acid (18:1) to form linoleic acid (18:2) while FAD7 is the enzyme to convert linoleic acid (18:2) to linolenic acid (18:3). Thus, higher transcript levels of FAD6 and FAD7 in the leaf with relatively lower levels in the bark led to a relatively lower concentration of oleic acid (18:1) and linoleic acid (18:2), and a higher level oflinolenic acid (18:3) accumulated in the leaf than in the bark.

## Conclusions

*W*. *ugandensis* has been used as a traditional medicine source in some African countries for a long history. Here we report the construction of a comprehensive transcriptome dataset (17.1G) derived from two different tissues (bark and leaf) by using Illumina sequencing technology. The database would accelerate functional genomic research on this plant. In particular, many candidate genes for structural enzymes in terpenoids and PUFAs biosynthesis were identified in this study, which would help people to understand the biosynthesis of terpenoids and PUFAs in *W*. *ugandensis*.

## Methods

### RNA extraction, cDNA library construction and sequencing

*Warburgia ugandensis* Sprague materials (bark and mature leaves) were collected from plants grown in the greenhouse of Wuhan Botanical Garden, Chinese Academy of Sciences, and immediately frozen in liquid nitrogen and stored at −80°C until use. Total RNA was extracted using the EASYspin plant RNA extraction kit (Aidlab Biotechlogies, Co., Ltd, China). RNA integrity and quantity were evaluated by the electrophoresis on 1% agarose gel and the detection on Agilent 2100 Bioanalyzer (S4 Fig). Equal amounts (about 5 μg) of RNA from three independent biological repeats for each sample were mixed together and then send to Shanghai Oebiotecth Corporation (Shanghai, China) for the sequencing by Illumina HiSeq2000 platform. The pair-end method was applied in this work, and the read length was 100 bp for each read. The quality of raw data was evaluated using the FastQC software (http://www.bioinformatics.babraham.ac.uk/projects/fastqc/), and low quality reads were discarded by use of NGS QC Toolkit (v2.3.3) software (http://59.163.192.90:8080/ngsqctoolkit/). First, removing all low quality reads with phred score less than 20 and read length shorter than 35 bp. Second, trimming bases having phred quality score less than 20 at 3’ end of the read. Third, trimming the sequences containing ambiguous ‘N’ bases, and reads shorter than 35 bp would be discarded. Then, the clean reads were assembled into contigs or unigenes using Trinity software (version: trinityrnaseq_r20131110) with the specific parameter set to “—seqTypefq—min_contig_length 200—JM 400G” and all other parameters set to default [39]. The raw transcriptome data was available at the sequence read archive (SRA) of NCBI with the accession number SRX970743.

### Functional annotation

The function of the unigenes was annotated by alignment of the unigenes with the NCBI non-redundant (Nr), SwissProt, and KOG (http://www.ncbi.nlm.nih.gov/COG) databases using Blastx with a threshold *E* value of 10^−5^. The proteins with the highest hits to the unigenes were used to assign functional annotations thereto. Gene ontology (GO) classification was performed by the BLAST2GO program [40]. The unigenes were mapped to the Kyoto Encyclopedia of Genes and Genomes (KEGG) database (http://www.genome.jp/kegg/) to annotate their potential metabolic pathways.

### Identification of terpene synthases in the *W*. *ugandensis* transcriptome

The terpene synthase N-terminal motif (pfam01397) was downloaded from the Pfam database (http://pfam.sanger.ac.uk/), and used as a bait to search against the *W*. *ugandensis* transcriptome. TPS domain sequences containing the pfam01397 motif were obtained using BLASTX with an E-value threshold of 10^−5^, and the full length coding unignes of TPS were predicted by ORF finder on line (http://www.ncbi.nlm.nih.gov/gorf/gorf.html). Several homologous TPS sequences in other plants [28, 30] including two *W*. *ugandensis* sesquiterpene synthase sequences (ACJ46047.1, ACJ46048.1), were downloaded from the Nr protein database and used as references to perform a phylogenetic analysis with our identified TPS. The phylogenetic tree was constructed by MEGA 5.0 using neighbor-joining method with 1000 bootstrap trials, and other specific parameters were set by default.

### Differential expression analysis of unigenes

The FPKM (fragments per kb per million reads) method was used to calculate the expression abundance of the unigenes [41]. The fold-changes of each gene expression between leaf and stem bark were evaluated by their ratio of the FPKMs. The DEGs were determined by the R package DESeq (http://bioconductor.org/packages/release/bioc/html/DESeq.html), which provides methods to test the differential expression by use of the negative binonial distribution and a shrinkage estimator for the distribution’s variance, with setting the threshold of FDR (false discovery rate) ≤0.05 and log2Ratio ≥ 1 [42]. Using a hypergeometric test (with *p* value < 0.05), the significantly enriched GO terms in DEGs were analyzed by comparing gene numbers for each certain GO term with the genome background of *W*. *ugandensis*. DEGs were also used in pathway enrichment analysis by mapping all the DEGs to KEGG database, the number of genes in each pathway was calculated and compared to the whole genome background by the threshold of *p* value < 0.05.

### GC-MS analysis

About 0.3 g fresh weight of *W*. *ugandensis* stem barks or leaves were collected, and immediately powdered in liquid nitrogen frozen. The plant tissues were extracted in 500 μl hexane by ultra-sonification for 30 min. The crude extracts were centrifuged at 12,000 g for 10 min, then 2 μl of the supernatant was analyzed on an Agilent 7890A GC machine (Agilent Technologies, Waldbronn, USA) and an Agilent 5975C mass selective detector. Separation was performed on an Agilent HP-5MS column (0.25 mm × 30 m × 0.25 μm), and the carrier gas was helium with a flow rate of 1.2 ml/min. The initial GC oven temperature was set at 50°C for 2 min, followed by a 5°C /min ramp to 120°C and a 30°C /min ramp to 240°C, and held at 240°C for 5 min. Compounds were determined by mass spectral comparison with the chemical database installed in the GC machine.

### qRT-PCR analysis

After treating with DNase I (Thermo, USA) to remove residual genomic DNA, 2 μg of total RNA was converted to the first-strand cDNA by use of SuperScript III reverse-transcriptase (Invitrogen, USA). The cDNA products were then diluted in 100-folds with deionized water prior to PCRs. The qRT-PCR reaction was performed on an ABI 7500 Real-Time PCR Detection System using a FastStart Universal SYBR Green Master Mix (Roche). The thermal cycling conditions were as follows: 95°C for 10 min for one cycle; at 95°C for 15 s, then at 60°C for 1 min for 40 cycles. Three independent biological replicates were performed for each sample. The relative mRNA levels were normalized with respect to inner control genes (actin) and expressed relative to the corresponding values of leaf (control), which were given an arbitrary value of 1. Primer pairs were designed using Primer 5 software, and the primer sequences were available on-line (S8 Table).

## Supporting Information

S1 Fig Length distribution of unigenes in the *W*. *ugandensis* transcriptome. (TIF)

S2 Fig Summary of differentially expressed genes between the bark and leaf. A: the number of the unigenes that are exclusively expressed in the bark or leaf, and the unigenes that are expressed in both tissues; B: the number of the differentially expressed genes (DEGs).
(TIF)

S3 Fig Putative structural genes involved inunsaturated fatty acid biosynthesis pathway. The values in the bracket indicate the number of unigenes in the corresponding gene families.KAS, ketoacyl-ACP synthase; KAR, ketoacyl-ACP reductase; HAD, hydroxyacyl-ACP dehydrase; EAR, enoyl-ACP reductase; SAD, stearoyl-ACP desaturase; FAD6, oleate desaturase; FAD7, linoleate desaturase; ACOT, acyl-ACP thioesterase.
(TIF)

S4 Fig The RNA integrity and quantity were evaluated by the electrophoresis on 1% agarose gel (A), and the detection of leaf (B) and bark (C) RNA by using Agilent 2100 Bioanalyzer. (TIF)

S1 Table KEGG pathway annotation of unigenes. (XLSX)

S2 Table Putative structural genes involved in terpenoid backbone biosynthesis pathway. (XLSX)

S3 Table The unigenes coding for putative terpene synthases in terpenoid biosynthesis. (XLSX)

S4 Table Unigenes annotated to be involved in unsaturated fatty acids metabolism. (XLSX)

S5 Table List of DEGs between *W*. *ugandensis* stem bark and leaf. (XLSX)

S6 Table GO enrichment analysis of DGEs. (XLSX)

S7 Table KEGG pathway enrichment analysis of DGEs. (XLSX)

S8 Table qRT-PCR primers used in this study. (XLSX)
