# Supplementary figures and images for: De Novo Transcriptome Analysis of Warburgia ugandensis to Identify Genes Involved in Terpenoids and Unsaturated Fatty Acids Biosynthesis
Source: PLoS One. 2015 Aug 25;10(8):e0135724. doi: 10.1371/journal.pone.0135724 (PMC4549110; doi:10.1371/journal.pone.0135724)

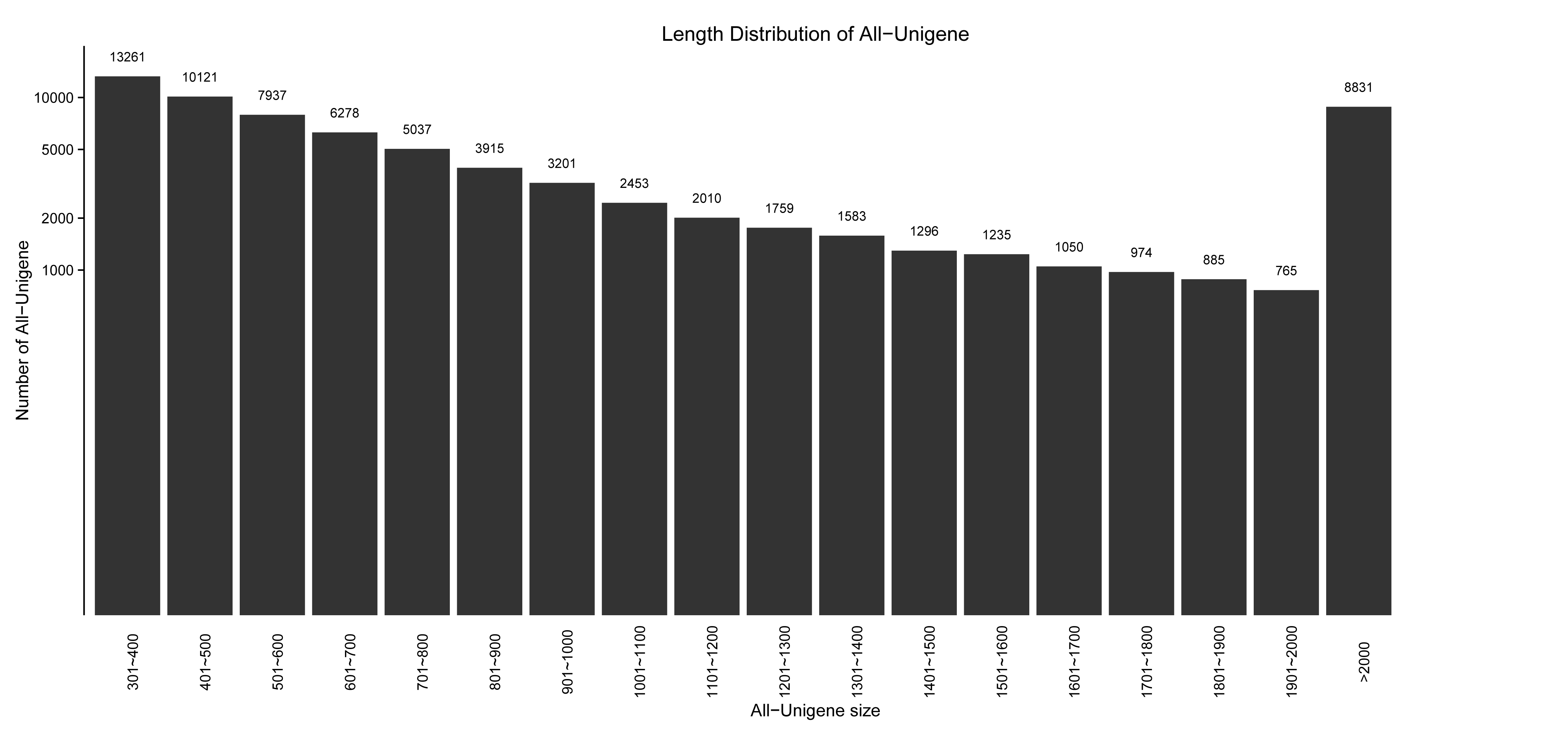

Supplement: S1 Fig — (TIF) [file pone.0135724.s001.tif]

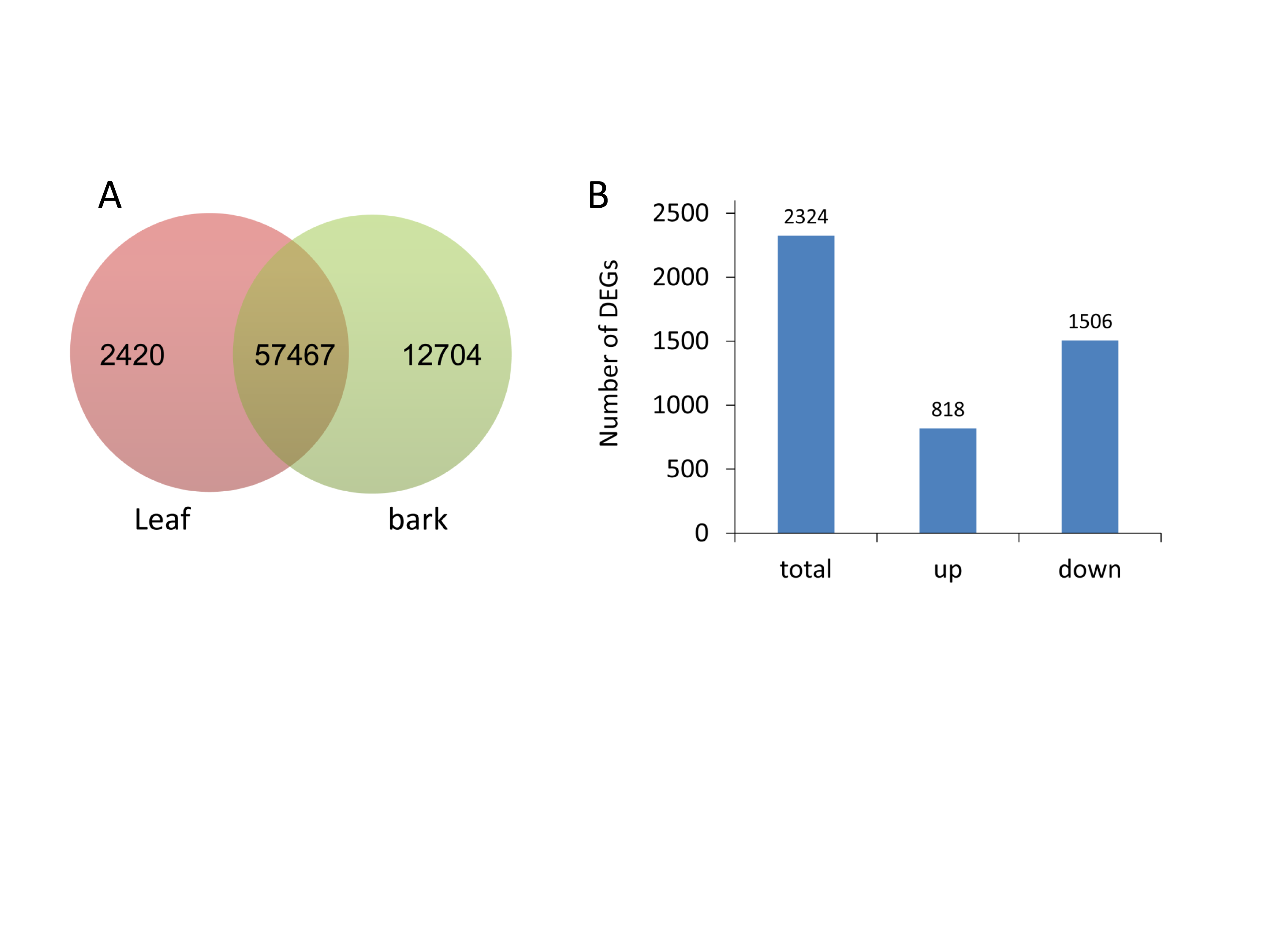

Supplement: S2 Fig — A: the number of the unigenes that are exclusively expressed in the bark or leaf, and the unigenes that are expressed in both tissues; B: the number of the differentially expressed genes (DEGs). (TIF) [file pone.0135724.s002.tif]

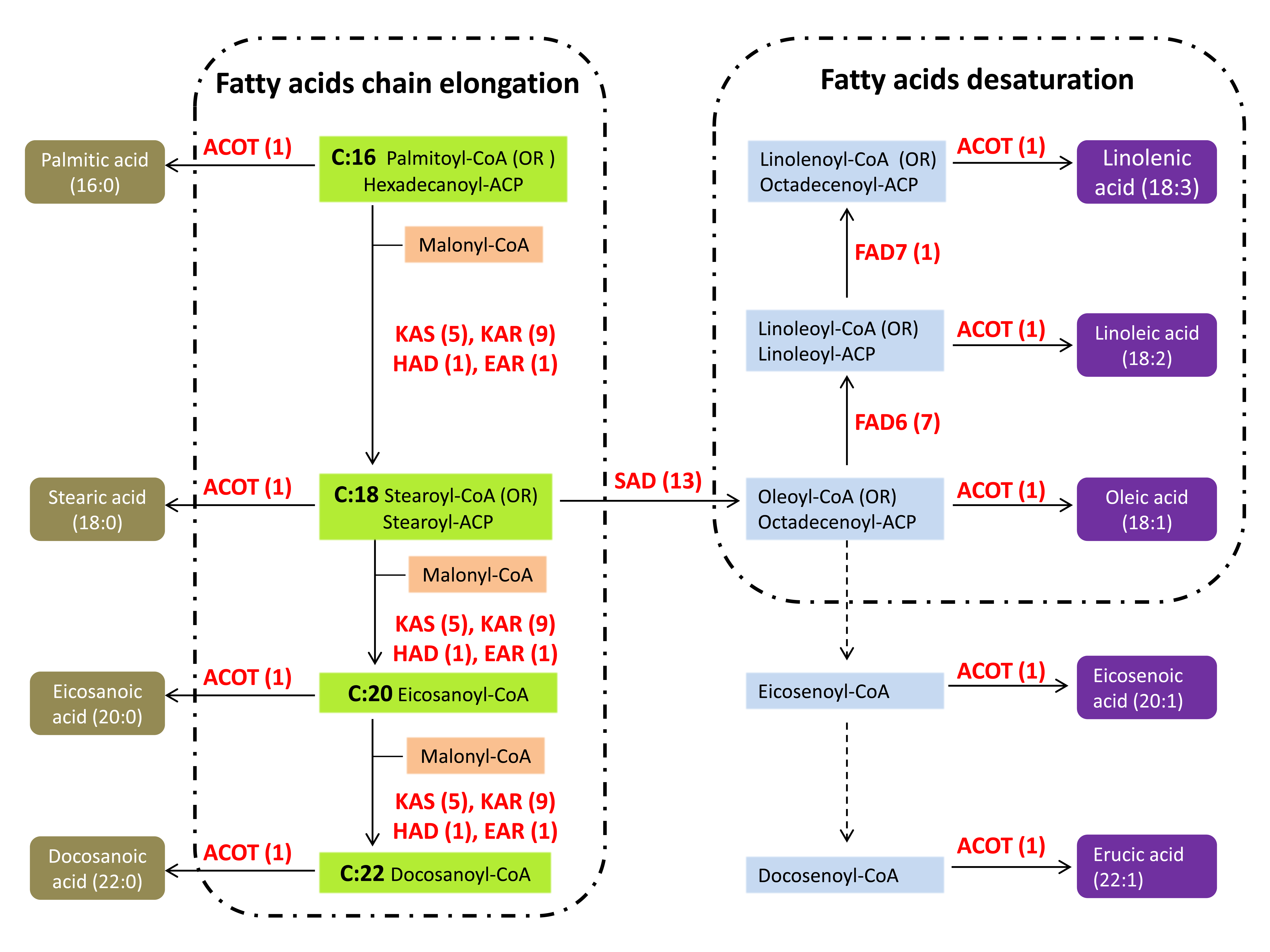

Supplement: S3 Fig — The values in the bracket indicate the number of unigenes in the corresponding gene families.KAS, ketoacyl-ACP synthase; KAR, ketoacyl-ACP reductase; HAD, hydroxyacyl-ACP dehydrase; EAR, enoyl-ACP reductase; SAD, stearoyl-ACP desaturase; FAD6, oleate desaturase; FAD7, linoleate desaturase; ACOT, acyl-ACP thioesterase. (TIF) [file pone.0135724.s003.tif]

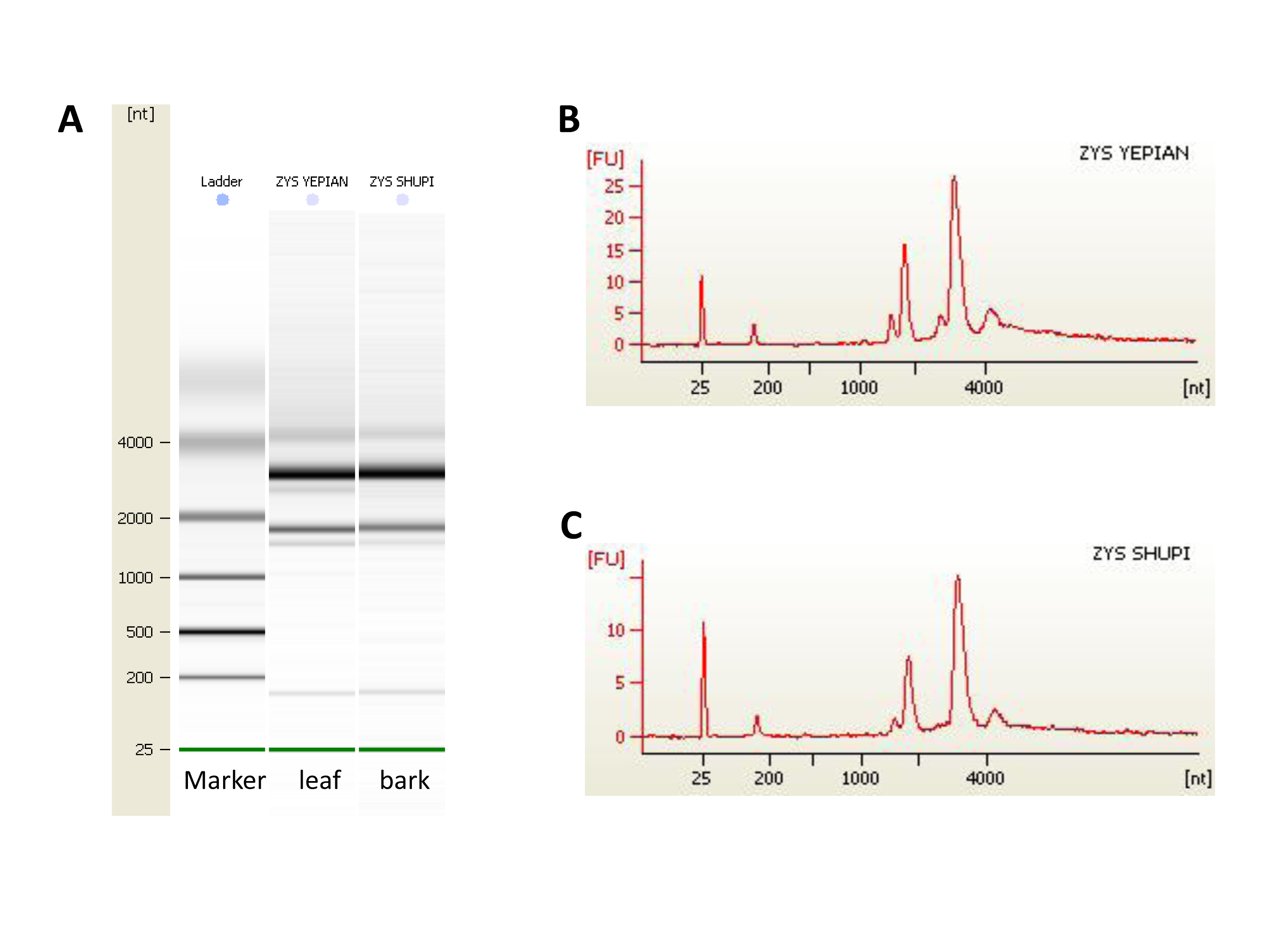

Supplement: S4 Fig — (TIF) [file pone.0135724.s004.tif]
